# ‘I wanted to talk about it, but I couldn’t’, an H70 focus group study about experiencing depression in early late life

**DOI:** 10.1186/s12877-020-01908-x

**Published:** 2020-12-07

**Authors:** Therese Rydberg Sterner, Synneve Dahlin-Ivanoff, Pia Gudmundsson, Stefan Wiktorsson, Sara Hed, Hanna Falk, Ingmar Skoog, Margda Waern

**Affiliations:** 1 Centre for Aging and Health (AgeCap) at the University of Gothenburg, Gothenburg, Sweden; 2 Neuropsychiatric Epidemiology Unit, Department of Psychiatry and Neurochemistry, Institute of Neuroscience and Physiology, Sahlgrenska Academy at the University of Gothenburg, Mölndal, Sweden; 3 Department of Psychiatry and Neurochemistry, Institute of Neuroscience and Physiology, Sahlgrenska Academy at the University of Gothenburg, Gothenburg, Sweden; 4 Region Västra Götaland, Sahlgrenska University Hospital, Psychiatry, Cognition and Old Age Psychiatry Clinic, Gothenburg, Sweden; 5 Region Västra Götaland, Sahlgrenska University Hospital, Psychosis Clinic, Gothenburg, Sweden

**Keywords:** Depression, Experience, Early late life, Focus group, General population, H70 study

## Abstract

**Background:**

Knowledge about experiences of depression among younger-old adults from the general population is limited. The aim was to explore experiences of depression in early late life.

**Methods:**

Sixteen participants in the population-based Gothenburg H70 Birth Cohort Studies (12 women and 4 men) who had reported a history of depression between ages 60–70 took part in focus group discussions (*n* = 4). Data were analyzed using focus group methodology.

**Results:**

The analysis resulted in the overall theme ‘I wanted to talk about it, but I couldn’t’. The participants expressed unmet needs of communication about depression with family, friends, and healthcare staff. Participants wanted to know more about the causes and effects of depression, available treatment options and how to avoid recurrence. Lack of knowledge was a source of frustration; trust in health care providers was diminished. Being retired meant that opportunities for communication with co-workers were no longer available, and this made it harder to break negative thought and behavioral patterns. Being depressed meant losing one’s normal self, and participants were grieving this. Thoughts of death and suicide were experienced in solitude; knowing that there was an escape could generate a feeling of comfort and control.

**Conclusions:**

Younger-old adults have expressed a need to talk about their experiences of depression. They would like to know more about available treatments, potential side effects, and how to avoid recurrence. Care providers also need to be aware there is a need for an existential dialogue about death.

**Supplementary Information:**

The online version contains supplementary material available at 10.1186/s12877-020-01908-x.

## Background

Depression in older adults is often overlooked and wrongfully dismissed as a part of aging. Late life depression is an escalating public health issue. As a result of increasing life expectancy worldwide, [1, 2] it is anticipated that the burden of depression on persons, their families and friends, and on society will increase. Currently, about 1–2% of men and 3–8% of women suffer from major depression at age 70 [3]. Corresponding figures for minor depression are about 5–7% in men and 8–12% in women. The association between depression and suicide is particularly strong in older adults, further emphasizing the importance of appropriate measures to decrease the incidence and prevalence of depression in older populations [4]. Despite these facts, knowledge about how older adults in the general population experience depression is rather limited.

A qualitative approach where personal narratives are in focus is warranted in order to gain a deeper understanding of how early late life depression is experienced. A meta-synthesis [5] of 13 qualitative publications exploring depression among adults aged 60 years and above identified four major themes: experiences [6–10], causes [6–11], recovery [6–10, 12, 13], and barriers to treatment [8, 11, 13, 14]. During the past decade, several studies have focused specifically on experiences of depression among older adults within specific cultural groups, including African Americans aged 55+ [15–17], Korean Americans aged 60–73 [18], and Hispanic immigrants in the United States aged 67–77 [19]. These studies showed cultural-specific manifestations and coping strategies for depression among older adults, emphasizing the need for cultural-appropriate interventions. Among studies that have focused specifically on help-seeking behaviors, symptom recognition, and recovery in relation to ageing, we note that two explore depression experiences among older adults aged 65–85 in Japan [20, 21], three from the United Kingdom involving persons aged 58–84 [22], 75+ [23], and 65+ [24], and one study set in Sweden in which all participants were age 87–88 [25]. In addition, experiences of depression in relation to masculinity and ageing were examined in two Canadian studies involving men age 55–80+ [26, 27]. These studies showed that traditional masculine ideals may be related to depression and adverse experiences of the retirement transition. With a few exceptions [6, 17, 18, 25], the majority of the above cited studies have included clinical samples from primary care or inpatient hospital settings, and most have involved mixed age samples, over an age range of up to four decades. Experiences of depression can be expected to differ over the aging process. While persons in their eighties and nineties may be coping with numerous age-related losses including loss of spouse, friends, health, functional ability and autonomy, those in their sixties may be just starting to transition into later life. In this study we wanted to focus on the latter group. We could identify only one population-based study with such a focus. In a sample aged 60–73, derived from the Memory and Aging Study of Koreans (MASK), [18] the authors reported that the majority of those with high depression symptom scores did not identify themselves as having depression, nor had they reported having had prior depressive episodes. Some of the MASK participants suffered from cognitive decline, which may have negatively affected their abilities to remember details of past experiences. The authors suggested that a linguistically and culturally relevant mental health service is needed to enhance help-seeking and symptom expression among vulnerable groups of older adults. This is important because perceptions and beliefs about depression can negatively influence older adults’ willingness to communicate depressive symptoms [24], and experiences of depression in early late life may differ depending on the cultural setting.

In the present study, we wanted to explore the experience of depression in early late life in a Swedish context. We applied focus group methodology in order to learn how younger-old adults from the general population experience depression with regard to perceptions, understanding, help-seeking and treatment, in order to identify potential unmet needs and knowledge gaps. Participants were recruited among 70-year-olds who reported in a population-based research interview that they had a depression at some point between the ages of 60–70.

## Methods

### Design

This study utilized focus group methodology [28] (i.e. when groups of people are interviewed collectively about specific topics) in order to capture an understanding of participants’ shared experiences of having depression in early late life. The method provides access to participants’ own language, concepts and concerns. The rationale for choosing focus group methodology was that group interactions tend to generate spontaneous and informal discussions among participants, which can empower and engage persons with limited influence [29], in this case, persons with recent experience of depression.

### Participants

**Fig. 1:**
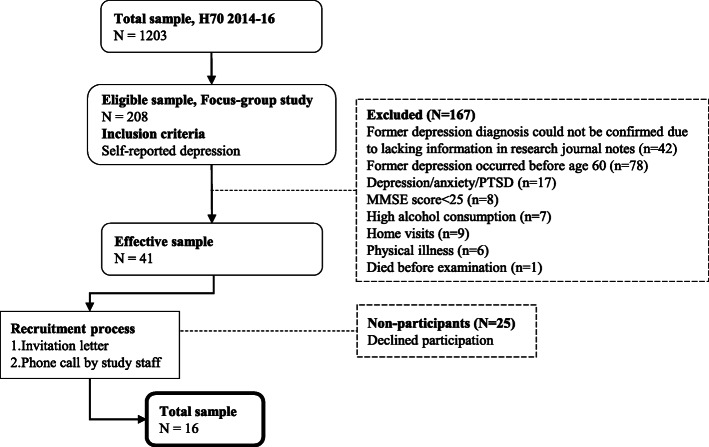
Sample flow chart

This study originates from the baseline examination of 70-year-olds in the population-based Gothenburg H70 Birth Cohort Studies (the H70 study) during 2014–16, previously described in detail [30]. The focus group sample was strategically selected from the H70 study sample (*n* = 1203). The sample flowchart is seen in Fig. 1. The basis for recruitment comprised self-reported information on having experienced at least one depressive episode between ages 60–70 (eligible sample *n* = 208). Research journals of eligible participants were audited, and a prior depression diagnosis was confirmed or refuted by a psychiatrist (MW) according to the Diagnostic and Statistical Manual of Mental Disorders Fifth Edition (DSM-5) [31]. Exclusion criteria included cognitive impairment (Mini-mental State Examination (MMSE) [32] score < 25), and depression secondary to severe alcohol use disorder, severe anxiety disorder, post-traumatic stress disorder, or severe physical illness. Further, persons with initial H70 study examination conducted by home visit were excluded as these were e.g. institutionalized, suffered from severe cognitive decline, or reluctant to leave their homes, which would hinder participation in the group interviews. The effective sample (*n* = 41) was contacted by mail, followed by a telephone call. All participants were asked about their current mood in order to exclude those acknowledging ongoing low mood. They also received written and verbal information about the study aim and procedures. Out of 41 eligible participants, 16 agreed to participate. Both homogeneity and heterogeneity were considered for each focus group. All participants shared the experience of depression between age 60–70. They had also participated in the H70 study at the age of 70. Heterogeneity was considered in terms of sex and country of birth.

### Data collection

#### Demographics

Demographic information about the participants was retrieved from the H70 study and included educational attainment, country of birth, having a partner/marital status, number of children and grandchildren, health care contact, and antidepressant medication (Table 1). Participants also completed the 9-item self-report version of the Montgomery Åsberg Depression Rating Scale (MADRS) [33] (possible range 0–54) at the time of the focus group discussions.

**Table 1:** Sample characteristics

**Age**, range (mean)	**71–72** (71.8)
**Demographics**, % (no. of cases/total cases)	
Female sex	**75.0** (12/16)
Primary education	**6.3** (1/16)
Secondary education	**43.8** (7/16)
Higher education	**50.0** (8/16)
Born in Sweden	**81.3** (13/16)
**Family**, % (no. of cases/total cases)	
Having partner	**81.3** (13/16)
Having children	**93.8** (15/16)
Having grandchildren	**87.5** (14/16)
**MADRS-S**^a^ score (range)	6–20
**Health care contact**	
During latest depressive episode	**100.0** (16/16)
**Antidepressant treatment**	
During latest depressive episode	**87.5** (14/16)
Now	**75.0** (12/16)

^a^Montgomery Åsberg Depression Rating Scale, self-report. Score at time of focus group discussion

#### Focus group discussions

The setting for the focus group discussions was the same outpatient clinic where participants had previously taken part in the H70 study. The participants were divided into four focus groups, with 3–5 participants in each. The groups were either all female or mixed in order to capture varying types of interactions during the discussion (focus group (1): 4 women; (2) 4 women; (3) 2 men and 3 women; (4) 2 men and 1 woman) [34]. Each focus group lasted approximately 1.5 h. A moderator (female) and assisting moderator (male) conducted the group discussions. The moderator began the session by clarifying that the participants were the experts in this context, as the research aim was to learn from their experiences. The discussion topic guide (see Additional file 1: Appendix 1) included open-ended discussion points about overall experience of depression, gender norms in relation to depression, coping, and depression treatment. The participants were encouraged to discuss openly, but at the same time to disclose only information which they felt comfortable about sharing with others. During the discussion, the moderator posed questions in order to deepen the discussions and ensured that all participants were given the chance to speak. All focus group discussions were audio-recorded.

### Analysis

The audio-recordings were transcribed verbatim and reviewed for accuracy in transcription. The analysis was based on a method developed by Kreuger and Casey [28]. The analysis was conducted in Swedish in order to stay close to the raw material, and was then translated into English. The audio recordings were listened to several times to get an overall sense of the data. The transcripts were carefully read independently by each of the two authors who conducted the analysis. Guided by the study aim, relevant themes were identified. The material was sorted by theme and emerging sub-themes were defined within each theme. Descriptive statements synthesizing, abstracting, and conceptualizing the data were extracted from the raw material. Finally, the categorized material was summarized and interpreted in order to provide an overall understanding. The analysis was conducted using NVivo 12 software.

## Results

### Overall theme: I wanted to talk about it, but I couldn’t

The overall theme ‘I wanted to talk about it, but I couldn’t’, summarized the participants’ discussions about experiencing depression in early late life. The participants wished for a deeper dialogue with their family and friends, and with their healthcare providers. They wanted to talk about their depression experiences, but were met with silence and left with unanswered questions. This created feelings of both fear and frustration, as the colors of everyday life faded and thoughts about the end of life emerged. The overall theme consisted of five specific themes: (1) Life in fading colors, (2) Struggling to communicate with others, (3) Fear and frustration of the unknown, (4) Approaching death – a double edged sword, and (5) Lack of trust in the professional management of depression. Table 2 provides an overview of theme structure.

**Table 2:** Summary of overall theme, themes, and sub themes

Overall theme	Theme	Sub theme
**I wanted to talk about it, but I couldn’t**	**1. Life in fading colors**	1.1. Grieving the loss of the old me 1.2. Suffer in silence
	**2. Struggling to communicate with others**	2.1 A new and unclear set of social rules 2.2 Normative attitudes about gender and depression
	**3. Fear and frustration of the unknown**	3.1. Fearing the depression 3.2. Not knowing the cause
	**4. Approaching death – a double edged sword**	4.1. Clashing expectations in the face of reality 4.2. Finding meaning in the threatening presence of death
	**5. Lack of trust in the professional management of depression**	5.1. Antidepressant medication - hero or villain? 5.2. Views on how the treatment should have been conducted

### Theme 1: life in fading colors

The colors of life were altered during depression. The previously vivid color palette slowly and continually changed towards shades of grey, generating a change in personality; nothing felt joyful anymore. The world continued outside while the study participants transformed from actively partaking in everyday life to becoming emotionally isolated observers. Communication with their surroundings faded due to social withdrawal, despite the need of wanting to talk about their experiences.

#### Sub theme 1.1: grieving the loss of the old normal me

As the depressive symptoms crept up on them, the faded self was slowly normalized. The perception of time deteriorated which generated a focus towards feelings of hopelessness; recovery seemed no longer an option. They got used to being depressed. Their sense of self was compromised, and they were saddened by the fact that depression made them feel like half a person. They became aware of their changed personality, and began to grieve the loss of their old normal, more colorful, selves.

#### Sub theme 1.2: suffer in silence

The participants withdrew and tried to cope with depression out of reach from the lights of their outside world. They suffered alone, and in silence. Friends and family ‘walked on eggshells’ when it came to the topic of depression; they kept their distance. Even though participants had social support from family and friends, it was never sufficient. Conversations were silenced or ended with lack of understanding. This included responses such as “get ahold of yourself” or “I don’t know how to handle you”. Participants experienced that family and friends grew tired of their low mood, and their fading self.

### Theme 2: struggling to communicate with others

The participants wanted to be able to talk more about what they were experiencing. Despite having knowledge about the importance of breaking their social isolation, it was hard to do single-handedly. Several barriers stood in their way. Being able to reach out to the outside world required an act of force, which was undermined by their lack of energy and motivation. In addition, unclear changes in the social norms of communication made it even harder to be part of the world outside themselves. Participants wished that their friends and family would have taken a greater responsibility in initiating dialogue, as it was difficult to actively ask for help. They expressed appreciation that the focus groups gave them a chance to share their experiences with others who had also had depression in early late life.

#### Sub theme 2.1: a new and unclear set of social rules

Depression changed the rules on how to communicate with the outside world. It became unclear what they should or should not say, or to whom they could tell, about what they experienced. The experiences of social support from spouses were mixed. Some had received support from their spouse, others did not, and a few expressed marital hardships during the depressive episode. Participants wanted to avoid having their children and grandchildren stepping in as ‘the adult’, to take care of their depressed parent or grandparent. It was especially hard to break negative and isolated everyday patterns after retirement, as regular communication with workmates was no longer available. The loss of everyday contact with co-workers became a barrier for reaching out. Participants feared being perceived as a burden on their former workmates, especially since the workplace had become a context to which they no longer belonged. Similarly, a fear of being a burden inhibited communication with friends and family.

#### Sub theme 2.2: normative attitudes about gender and depression

It was more difficult for men than for women to talk about their feelings. This was how they were raised; it was not something that they thought about in relation to their own experiences of depression. Men perceived that it was not as socially accepted for a man to feel sad, cry and express depressive symptoms as it was for a woman. These normative attitudes towards gender were a barrier towards talking about depression to friends and family, as well as to healthcare staff. Women expressed having strong social networks in and outside their families. Although the traditional gender roles made women more prone to talk about their problems compared to men, participants felt that others expected them to solve their own problems, i.e. deal with their depression.

### Theme 3: fear and frustration of the unknown

Participants feared that the depression would never pass. This fear also included the possibility of recurrence after treatment. They expressed a frustration towards the painful uncertainty of not knowing the cause of the depression and its potential consequences for body and mind. They wondered if this was what the rest of their lives was going to be like. These fears and frustrations were enhanced by the fact that no one was able to answer their questions.

#### Sub theme 3.1: fearing the depression

One of the most difficult parts of being depressed was the fear of never recovering. They did not see a way out, and it was hard to believe in the dawn of a bright future. Once the depressive symptoms started to remit, fear of never recovering was replaced by fear of relapse. Depressions that ‘came from within’ were considered a larger threat, as participants believed that the risk of recurrence was greater, compared to depressions sparked by external factors. They also feared that depression might generate negative physical and mental effects. The fear of the unknown never went away, and there was a desperate need for an answer to the question ‘will it [the depression] ever truly pass?’

#### Sub theme 3.2: not knowing the cause

There was a substantial frustration in not knowing the cause of depression. Participants were also frustrated about not receiving answers from health care providers regarding the possible physical and mental effects of depression. The participants spent a lot of time trying to figure out potential causes and reasons for recurrence. They felt relieved if they could point to a specific life event as the probable cause. It was easier to cope with depression when one could blame an external factor. If the cause of depression was unknown, it was described as ‘coming from within’ which generated frustration and self-blaming.

### Theme 4: approaching death – a double edged sword

Thoughts about death were often present; death was moving closer due to aging. The struggle of approaching death was considered to be a potential cause for depression, but on the other hand, thoughts about death were considered to be caused by depression. Suicide was sometimes perceived as being a potential source of rescue, a means to terminate the misery. The lack of communication with friends, family or health care providers during these darker shades of depression led to an inner struggle that was experienced in solitude. It was hard to find meaning in life during this time. Approaching death due to aging was considered a threat, as well as a potential rescue and a way out of the misery of depression.

#### Sub theme 4.1: clashing expectations in the face of reality

The expectations of a ‘golden’ post-retirement period were thrown aside when depression challenged the prerequisites for well-being. Participants told of a clash between positive expectations of later life and the negative reality of depression. It was difficult to deal with somatic illnesses, since the aging body became a reminder of mortality. The awareness of coming closer to the end of life became more apparent. Their bodies started to fail but their souls remained young, creating an inner discrepancy. Many faced burdensome social situations involving care of family members, and worries about the well-being of children and grandchildren. As a friend, parent or grandparent, there was a wish to be needed. They were saddened when depression rendered them unable to meet the need of others, and sometimes felt that being needed was too burdensome.

#### Sub theme 4.2: finding meaning in the threatening presence of death

Many prior activities were no longer meaningful. The participants did not feel as needed by others as before. Death of friends, relatives or neighbors reminded them that they could be next in line. This made it harder to see the point of living while struggling with depression, especially when it felt like this state would last forever. In secret, they made plans for their own funeral or suicide. Thus, death was no longer perceived as a threat, but rather a way out of misery. This created a feeling of being in control. Reasons for not choosing death over life included unwillingness to leave their children and grandchildren, or putting the needs of family members first. Some simply refused to give up and be defeated by depression. Participants avoided sharing these thoughts with their loved ones in order to protect them.

### Theme 5: lack of trust in the professional management of depression

Trust towards the services for the management of depression was lacking. Participants described an unmet need for dialogue with health care staff during help-seeking, as well as during and after treatment. The duration of personal contact was limited; at times it was non-existent. Participants questioned whether health care staff knew enough about causes of depression, about how it affects the body and brain, and how to avoid recurrence. If depression would recur, they did not trust that they would be referred to the proper care facility, they doubted that they would receive person-centered treatment, and they questioned whether there would be enough hospital beds or available health care staff. This was rooted both in own experiences, and in media coverage of budget cuts for psychiatric health care in Sweden. Participants expressed that one must be severely ill in order to receive ‘real’ health care for depression.

#### Sub theme 5.1: antidepressant medication – hero or villain?

A dualistic attitude towards antidepressant medication was apparent. Participants related their fears of adverse side effects or addiction. This was a barrier for accepting treatment. On the other hand, antidepressants were seen as a last chance to escape the darkness of depression. The upside of treatment was related to the experience of finding one’s way back to life from a depressed state. They felt reluctant about stopping antidepressant treatment due to fear of depression recurrence. The downside of antidepressant treatment involved the unspoken side effects including loss of libido, loss of both happy and sad emotions, and feeling like a zombie. Lack of communication about side effects made them feel neglected and unprioritized.

#### Sub theme 5.2: views on how the treatment should have been conducted

Participants felt that they had not received the type or amount of treatment they thought necessary. They wished for better communication with health care providers before, during and after pharmacological treatment. This would have made them feel more prepared for potential side effects. No one talked to them about their hardships in finding meaning in everyday life, or about their thoughts of death or suicide. They were frustrated by the lack of access to psychological interventions. Compared to pharmacological treatment, attitudes towards psychotherapeutic alternatives were more positive. However, privately funded alternatives were costly. This limited the availability to long-term psychosocial treatments. They wanted to participate actively in treatment decisions, which would have required more information about the pros and cons of various treatment options. Information on how one could prevent depression recurrence was considered a top priority.

## Discussion

### Main findings

The overall theme ‘I wanted to talk about it, but I couldn’t’ summarized the experience of having early late life depression. The participants expressed unmet needs of communicating with their loved ones, as well as with health care staff. Further, they described a lack of trust in their health care for depression. They were frustrated with not knowing possible causes and effects of depression, not being informed about treatment options and not knowing how to avoid recurrence. Having depression in the post-retirement period meant that it was hard to break negative and isolated everyday patterns since the communication with co-workers was lost. Depression was not perceived as a normal part of aging. The participants grieved the loss of their old normal selves. Thoughts of death and suicide were experienced in solitude as health care providers did not seem interested or prepared to discuss these issues.

### The need for an existential dialogue during early late life depression

We found that, together with barriers of reaching out to others [15, 16, 18] and social stigma after disclosing having depression [15, 16, 18], our participants emphasized a need to be needed. Although sometimes incapable of meeting the needs of others during depression, it was important to still feel needed in their roles as friends, parents or grandparents. This is in line with the results of others [6, 21], who also pointed out the need to feel needed by society [21]. Older adults with depression have expressed that not feeling needed generated thoughts that no one would care if they died [21]. Despite the relatively young age of the participants, there was a need to have an existential dialogue about aging and death during their depression. Death was perceived not only as a threat, but also as a potential source of rescue from depression. This was in line with our previous finding that some older adults attributed their suicide attempts to a need to escape [35].

### Need for knowledge

Our findings indicate that persons who experience depression in early late life want to improve their depression health literacy. Unlike numerous other studies reporting that limited knowledge and recognition of depression may prevent older adults from seeking help [15–18, 22], the issue here was not lack of knowledge regarding the recognition of depression symptoms. All had acknowledged a past period of depression during the initial H70 examination, and all were cognizant of the symptoms of the illness and the consequences it had on their daily lives. Participants’ needs for increased knowledge about depression involved possible causes and effects of depression, non-pharmacological treatment options, and how to avoid recurrence. Further, they wanted to know how they could improve communication about depression-related issues with their families and friends, as well as with health care professionals. Regarding the latter, information on the pros and cons of different treatment options was sorely needed. This has been reported by others [16–18, 23], and is a prerequisite for patient participation in treatment decisions. Our study participants feared that antidepressants would become addictive, a perception also found by others [14, 15, 19, 36]. They also felt reluctant to take antidepressants due to potential adverse side effects. Despite these fears, many expressed that antidepressant treatment had been their only way out, and had given them their lives back. In a study focusing on somewhat older adults who attempted suicide (mean age 80), the necessity of antidepressant treatment was perceived to outweigh concerns related to adverse side effects [36].

### Unmet health care need

Our participants expressed feeling neglected both during and after depression treatment. They expressed a lack of trust regarding the professional management of depression. Consultations were short and information limited. Our results are in line with those of others who have reported low treatment expectations [6, 15, 16, 19, 22, 23] and uncertainty regarding the level of knowledge about late life depression among healthcare staff [16]. Swedish healthcare has undergone dramatic changes during the past decades [37], and our participants were aware of the lack of access to specialized mental healthcare, from both own experiences and from media reporting. However, this unmet need for mental healthcare is a global phenomenon [38]. In line with our participants’ perception of not receiving all necessary treatments, several national reports have stated that older persons suffering from mental illness are in fact not receiving the healthcare they need [39–41]. It has been suggested that antidepressants are prescribed to older adults by default, due to lack of time for discussion about other treatment options [24]. Our participants expressed a frustration over the lack of psychotherapeutic options for depression treatment. The Swedish National Board of Health and Welfare acknowledges that depressed older adults have less access to specialized care and psychotherapeutic alternatives compared to their younger counterparts [40, 41]. Our participants did not trust that they would be prioritized by health care services if depression recurred. This may have adverse effects for future help-seeking behavior, lowering the chances of receiving treatment. Further, for young old adults who are unable to recognize they are suffering from depressive symptoms, unmet needs and negative perceptions of health care are likely even more problematic.

### Gender and generation-related barriers for communication

We found masculinity-related barriers towards talking to others and expressing depressive symptoms. This may reflect the stereotypical preconception that disclosing low mood is feminine, which may be stigmatizing for men with depression [42]. However, in other studies among older persons, both women [6] and men [15] with depression considered themselves to be weak if ‘admitting’ to having depression. Further, we found that women had been more prone to talk about their feelings compared to men, and that they had strong social networks within their families and beyond. This adds to previous studies suggesting that women and men create different kinds of social relationships, which affects their social support [43]. Women tend to have larger and more intimate social networks, which may be beneficial in relation to communicating about low mood. The majority of the study participants reported having a partner. Some had experienced support from spouse, while others did not, and a few indicated marital hardships before and during the time of depression, which reflects previous studies showing that having a partner may have protective and supporting effects against depression (e.g. social/economic support, lower feeling of loneliness) [44, 45], but it may also be a risk factor for depression (e.g. emotional, physical or financial abuse, or marital dissatisfaction) [46, 47]. We also found a generation-related barrier towards communicating about depression. Participants declined to talk about their depression with their children or grandchildren. This was related to fear of being a burden; they wanted to avoid that their offspring would need to step into the role as ‘the adult’. Another reason for being reluctant to share experiences was expressed by older adults hospitalized for depression; they believed that the pains of aging and depression cannot be understood by those who have not experienced them personally [21].

### Depression not a normal part of early late life

We found a clash between expectations and reality regarding wellbeing after retirement, due to depression. Hence, our participants did not consider depression to be a normal part of aging. Instead, they were grieving the loss of their old normal selves, described by others as having spiritual pain [21] which reflects losses and challenges in relation to aging. Previous studies have reported that lower mood was anticipated by younger-old adults in their late 60s [15], frail older adults in their late 70s [23], and older-old adults in their late 80s from the general population [25]. Having low mood was normalized alongside of declining physical health [22], and was ‘simply something to live with’. Hence, it was perceived to be inappropriate to report low mood to health care staff [48]. Discrepancy between these previous results and those of our own study may be due to age and socio-cultural differences. Our participants did experience aging and declining physical health, but their disinclination to talk about their low mood with former workmates, friends and family, was related to the fear of overstepping and being a burden on others. Early late life and entry into old age is manifested through the retirement process, shaped by societal norms and expectations of older adults and their societal role. Studies among male Canadians showed that retirement was perceived as a loss due to masculine ideals of social and breadwinner status [26, 27]. This was suggested to be linked to both depression and suicide among men. In Sweden however, retirement has been shown to have limited [49] or positive [50] effects on well-being. In addition, findings from the H70 studies [30] have showed that successive birth cohorts of 70-year-olds are healthier and more active compared to prior generations [3, 51–55], with a higher anticipated life expectancy [56]. Therefore, our participants’ expectations of post-retirement health status may differ from those of earlier generations, as older adults of today are exposed to societal expectations of successful aging. This was indicated when the participants expressed a clash between their expectations of their post retirement phase of life, and the reality they experienced during their depression. If the normative retirement is anticipated to be a positive new phase of life, we need to consider the effects for retirees with non-normative experiences and the impact of maladjustment on physical and mental health.

### Clinical relevance and implications for prevention

The results of this study may have important implications for primary care and mental health professionals who work with depressed younger-old adults. Older adults, in comparison to younger adults, lack access to specialized mental health care and are more often prescribed pharmacological treatment, and access to adequate psychotherapeutic and psychosocial alternatives is limited. Primary care is the typical point of contact for older adults, involving both somatic and mental health. Hence, a major challenge for health care providers is to address the mismatch between health care policies and the actual care offered. Knowledge about late life depression needs to be increased among clinicians in primary care, with improved possibilities for referral to specialized care when indicated. Better communication skills are needed- both on the part of the depressed persons themselves and their health care providers. The accessibility of psychological treatment should be increased for mild, moderate and severe depression [57]; especially CBT [58, 59]. A more person-centered approach could include active participation in treatment decisions involving both psychosocial and pharmaceutical interventions, as well as attention to existential issues. More information about different treatment options and their potential effects, including side effects, should be provided. The expressed need to talk about depression may partly be met through discussion groups for younger-old adults to share experiences, or by including family members or friends in the treatment process. Our participants showed great appreciation for getting the chance to talk to peers with experience of depression, which expands on findings from a study involving older adults (age 58–84) [22]. However, some older adults might hesitate to join a support group, fearing it might negatively affect their mood [23]. A pilot study involving group discussions with structured reminiscence and a problem-based approach showed reduced depressive symptoms in older adults [60]. Creating meeting platforms with targeted group discussions has shown promising results in a Swedish pilot project aiming to prevent mental illness for older adults (age 78–93) in the general population [61]. This may also be beneficial for younger-old adults as a preventive action against depression.

### Strengths and limitations

Strengths of this study include that self-reported depression was evaluated by a psychiatrist prior to study inclusion. The first author moderated the focus group discussions and was the leading researcher during the analysis, which is considered advantageous in focus group methodology [28, 29]. We further accounted for trustworthiness by having two researchers conducting the analysis, interpreting the results together with co-authors. The focus groups enabled the participants to verbalize and share their lived experiences, generating an in-depth understanding of subjective experiences of early late life depression. There are also some limitations. The sample displayed a higher proportion of those having a partner (81.3%) compared to the research study population from which it was derived (70.7%). Having a partner may have increased the probability of accepting to participate in our focus group study. Issues involving communication and social support might have been even more prominent had our own participants been more representative of the H70 study population. Due to the small effective sample together with a low acceptance rate, the number of conducted focus groups was limited to four. This may negatively have affected data saturation and transferability of results. However, some suggest that four to five focus groups are sufficient when working with specific target groups [29], which is the case here. The number of participants in each focus group was small. There are no strict criteria in previous literature, in which the suggested number range between four to 12. As small groups can be very dynamic, the outcome depends more on the level of participants’ involvement than on the number of participants [29]. However, since few men participated, we were unable to conduct focus group discussions with men only. Another consideration is that while potential participants denied low mood at the initial telephone call, not all were in total remission on the day of the focus group interview, which took place 1–2 weeks later. Ongoing symptoms of depression might have colored the input of some participants. A final limitation is that sharing experiences from past episodes of depression may suffer from recall bias.

## Conclusions

This study contributes with new empirical knowledge of how younger-old adults from the general population experience depression. Our findings highlight that younger-old adults want to talk about their experiences, and to increase their health literacy regarding available treatment options for depression, possible side effects from pharmacological treatment, and preventive actions to avoid recurrence. Already early in late life, there may be a need for an existential dialogue about death while experiencing depression.

## Supplementary Information

**Additional file 1: Appendix 1.**

## Data Availability

Not applicable. This is a qualitative study and the data generated in the study are not available in line with information provided to the participants in the informed consent and all attempts would be made to maintain confidentiality. The raw data may be available upon reasonable request from the corresponding author.

## Abbreviations

DSM: the Diagnostic and Statistical Manual of Mental Disorders
H70: the Gothenburg H70 Birth Cohort Study
MADRS: the Montgomery Åsberg Depression Rating Scale
MASK: the Memory and Aging Study of Koreans
MMSE: Mini-mental State Examination.
