# Supplementary material for: ‘I wanted to talk about it, but I couldn’t’, an H70 focus group study about experiencing depression in early late life
Source: BMC Geriatr. 2020 Dec 7;20:528. doi: 10.1186/s12877-020-01908-x (PMC7720563; doi:10.1186/s12877-020-01908-x)
Supplement: Supplementary file 1 — Additional file 1: Appendix 1. [file 12877_2020_1908_MOESM1_ESM.docx]

**Appendix 1.**

**Focus group discussion guide**

- Overall experience of depression

(instruction: describe latest depressive episode / what was considered to be the toughest/hardest to handle / perception of social support and/or prejudices)

- Gender norms in relation to depression

(instruction: experiences of late life depression as man/woman / experiences of gender-related attitudes towards depression)

- Coping

(instruction: describe how they made it through the day / describe the utilization of coping strategies)

- Healthcare treatment

(instruction: describe why (or why not) they sought help from healthcare / experiences of healthcare for depression / experiences of treatment
